# Editor’s Book Table

**Published:** 1871-09

**Authors:** 


### Editor's Book Table.

[NOTE.—All works reviewed in the columns of the CHICAGO MEDICAL JOURNAL may be found in the extensive stock of W. B. KEEN & COOKE, whose catalogue of Medical Books will be sent to any address upon request.]

*A Physician's Counsel to Woman in Health and Disease.* By WALTER C. TAYLOR, A.M., M.D., author of "Gynæcological Notes;" "A Physician's Counsel to Man in Health and Disease," etc., etc. Springfield: W. J. Holland & Co. 1871.

The present seems fated to be the age of "femmes savantes," but without its Molière to teach it "Qu'un sot savant est sot plus qu'un sot ignorant." While we unite with the author, and "the most eminent physicians," to whom he alludes in his preface, in "deploring the ignorance which produces the vast amount of disease amongst women," we must be permitted to express a doubt, as to the value of such modes of correcting the difficulty, as the one which he has devised. "Counsels to women in health and disease by a physician," we most heartily approve, but such counsels would possess much greater value when imparted by the personal medical adviser of the woman, who should frame his counsels upon the individual peculiarities, capacities and necessities of the patient, rather than upon those of the sex at large. Such counsels, we believe, would not only be much more beneficial, but also much less injurious, than when conveyed by wholesale in book form. The latter require, for their consideration and application, a large share of that quality of which sick women generally possess a plentiful lack, i. e., judgment.

Moreover, the greatest want of humanity, in this era of its history, is want of faith, a want which underlies by far the greater portion of the evils which afflict society. A necessary outgrowth of this is a feverish effort at self-government, self-control, self-guidance; and to this morbid spirit of self-sufficiency, all such quasi instructors contribute. The tendency of books such as the volume before us is simply to shake public confidence in the medical profession, by the cultivation of sciolism; and to demoralize society by exposing to the ignorant and pure, a knowledge of human imperfections which should be forever hidden in the memory of the physician and in the consciences of the guilty.

But, while we condemn the publication of such books in general, we must concede that the present volume is evidently conceived in good faith, and written with much care; what is said is all very true, and well expressed, by an author actuated by an honest and earnest desire to benefit humanity. The book, in itself, is as far from objectionable features as any book of its character can be; and if women must be learned, let them by all means read this, rather than that other, bearing as its imprimatur the hoof-print of its satanic instigator.

We have no desire to repress the inquiring mind, in the pursuit of that knowledge which is profitable; but "There is a knowledge which is forbidden because it is dangerous." Remember the apple! Remember Cupid and Pysche; remember Cornelius

Agrippa's library; remember Blue Beard! "Curiosity," says Fuller, "is a kernel of the forbidden fruit, which still sticketh in the throat of a natural man (woman) sometimes to the danger of his choking." If the binder of the book had placed the pages in consecutive order, it would have facilitated the reading of it.

W. H.

*On the Cellular Structure of the Red Blood Corpuscle.* By Joseph G. Richardson, M.D., Microscopist to the Pennsylvania Hospital, Philadelphia, Pa. Reprinted from the Transactions of the Royal Microscopical Society, etc. etc.

This little pamphlet, originally published in the Transactions of the American Medical Association, contains Dr. Richardson's conclusions from his own observations upon red-blood cells, and which differ essentially from those of other eminent observers. Without attempting to enter into the merits of the discussion here, we will say that the author's observations, not only upon this subject but also upon white-blood cells, are well worthy of careful examination by every student of physiology.

We believe Dr. Richardson to be not only a skillful but an honest observer, who constructs his theories upon his observations, and not, like some whom we might name, his observations upon his theories; for to many the cloud will be "like a camel, indeed"—or "backed like a weasel,"—or, indeed, "very like a whale," according as one or the other fits the pet theory. W. H.

*A Treatise on Diseases of the Nervous System.* By Wm. A. Hammond, M.D., Professor of Diseases of the Mind and Nervous System, and of Clinical Medicine in the Bellevue Hospital Medical College, Physician-in-chief to the New York State Hospital for Diseases of the Nervous System, etc. New York: D. Appleton & Co.

It sometimes seems strange that authors write prefaces, so frequently are they irrelevant, and apparently having more reference to the form and appearance than to the subject-matter of the work. Dr. Hammond's preface is an exception in this regard, and one is rarely found which so clearly designates the character of the book as his. So that the reader, who wishes a peep in advance, may be sure of no after disappointment in the perusal of the work.

Dr. Hammond has, already, as an original observer, and an accomplished physiologist and psychologist, received such deservedly high commendation from every direction that additional praise would be supererogatory; but upon one point especially does he deserve praise, for his efforts, and successful efforts, to elevate professional literature to a sphere to which it dared scarcely aspire a few years ago. He has been one of the most prominent of the initiators of classical medical literature in America, and the appearance of such scholarly writers, gives good ground for the hope that the day is passing, if not already passed away, when reputations, as authors too, God save the mark! can be built upon published compilations, from standard authors, travestied into language which should shame a school-boy. The present work may, without invidious comparison, be not inappropriately termed the Doctor's "magnum opus,"—and if it has any especially distinctive characteristic, it is, we think, comprehension. Its scope is wide, its classification clear, and its details concise.

Free from useless verbiage, and obscurity, it is evidently the work of a man who knows what he is writing about, and knows how to write about it.

The subject-matter of the work is divided into three principal and two subsidiary chapters, treating respectively of the diseases of the cerebral, spinal and cerebro-spinal systems, of nerve-cells and of peripheral nerves. Prefixed to these, is an introductory chapter, in which are described "the instruments and apparatus employed in the diagnosis and treatment of diseases of the nervous system."

Surgical diseases and injuries are not embraced within the comprehension of the work; nor has the author allowed his diagnostic acumen to be misled by that hobby of routinists, that phase of typhus with secondary meningeal inflammation, ycleped cerebro-spinal-meningitis, which three or four years ago carried off so many victims intellectually as well as physically.

The author's pathological classification is strictly physiological, and the normal point of departure is never lost sight of in the morbid perversion of vital processes.

Is the hope Utopian, which would look to the elimination of this pseudonym (pathology) from medical nomenclature?

The system of classification is simple, and based principally upon disturbances of the relations of the nervous system and its membranes with the circulatory system, and comprehends in one uniform plan its cerebral and spinal subdivisions more particularly.

The author's views upon cerebro-spinal diseases are characterized by a much more than ordinary degree of clearness, and give promise of a future, pregnant with hope not only for sufferers from, but for students of these hitherto opprobria-medicorum.

Drawn largely from standard authorities, and sources, medical, philosophical and literary, the subject-matter has been re-cast in the author's own mind, and mingled therein with original observations and deductions, the work bears the impress of originality, and is essentially his own.

In reading that portion of the work treating upon perturbations of cerebration, purely, when having reached the boundary of the physical world the author glances into the realm beyond, it is gratifying to perceive that he has to some extent receded from the extremely materialistic standpoint which he formerly occupied.

Thus, while it can scarcely be admitted that "by mind, therefore, we understand a force *developed* by nervous action and especially by the action of the brain"—although it is undoubtedly thus *manifested*, the language is considerably nearer to physiologico-psychological truth than that "the brain secretes mind," (Journal of Psychological Medicine, January, 1869, page 168.) Neither can it be admitted that "Mind is a compound force *evolved* out of the brain," if by *evolved* (p. 330) generated (p. 324) is to be understood, as the context would seem to indicate. There is no mystery involved in the relations of mind, so-called, and brain, to him who boldly takes his stand upon the basis of a-priori facts, *i. e.*, faith, which all must do eventually, however long the chain of intermediate causes may be spun; and no greater *petitio principii* is made by him who assumes the a-priori existence of mind, than by him who admits no greater assumption than protoplasm.

But as the author touches very lightly upon this phase of nervous manifestations, courtesy demands that his reticence be imitated, in the hope that he will voluntarily recede still more thoroughly from his doubtful ground, and boldly, as he once did, reject the delusion of materialism.

In concluding what must, of necessity, be a very superficial review of such a work, perhaps no better advice could be offered to members of the profession interested in kindred subjects, than that which the reviewer has himself acted upon, "Buy it, study it carefully, from title page to index, and lay it aside to be criticised at leisure." W. H.

PAMPHLETS.

*The Lens; a Quarterly Journal of Microscopy and the Allied Natural Sciences: with the Transactions of the State Microscopical Society of Illinois.* Chicago, July, 1871. Vol. I, No. 1.

PROSPECTUS.

The State Microscopical Society of Illinois proposes to issue on the first of October, next, the first number of a Scientific Journal, to be called THE LENS. While THE LENS will be devoted chiefly to the interests of Microscopical Science, no communication of value, relating to any department of Natural History, will be excluded. Without trespassing on the fields so ably occupied by *Silliman's Journal* and the *American Naturalist*, the only publications of like character in the United States, the pages of THE LENS will contain:

1. Original contributions consisting of papers read before some Scientific Society, or communicated directly to the Journal;
2. Original papers, elaborate or otherwise, illustrative of the *Natural History* of the Mississippi Valley, and the Far West;
3. A comprehensive *resumé* of the latest foreign inquiries, and critical reviews, with brief notices of the latest microscopical publications in this country and Europe;
4. Descriptions of all new forms of Microscopes and Microscopic Apparatus; and
5. Correspondence on matters of Histological controversy.

Contributions requiring illustration will be accompanied by carefully drawn plates, and the text will be printed on good paper in clear and legible type.

THE LENS will be thoroughly scientific, advanced and comprehensive, and will be issued under the auspices, and in the interests of this society. Each number will contain at least 48 pages of reading matter. Terms, $2 per annum, *in advance.*

In view of the fact that there are so few journals published in this country, in these interests, the committee hopes for the strong support, both contributional and financial, of all lovers of Science.

Correspondence relating to the business management of the journal should be addressed to CHARLES ADAMS, Secretary of the publishing committee, 1000 Michigan avenue, Chicago. All other communications to the editor, S. A. BRIGGS, 177 Calumet avenue, Chicago.

E. H. Sargent, Charles Biggs, Charles Adams, Publishing Committee.

Officers of the Society: H. A. Johnson, M. D., Pres.; S. A. Briggs, 1st V. Pres.; H. H. Babcock, 2nd V. Pres.; Geo. M. Higginson, Treasurer; O. S. Westcott, Sec.; Charles Adams, Cor. Sec.

The above indicates the initiation of a movement in the right direction. We welcome it most cordially, and request for it the hearty encouragement and co-operation of other professional and scientific journals.

We are glad to see that the Microscopical Society, under its present efficient administration, so fully appreciates the importance of its mission as a public educator, as to desire to extend its sphere of usefulness and draw from other and foreign sources additions to its own stores, with which to feed the cravings of the public appetite for information in natural science. The society, although as yet only in process of crystallization as a scientific organization, has accomplished indirectly much good, by cultivating a taste for microscopic investigation, and although, it may be objected by carping critics, that with most, the instrument is merely a source of amusement, still, we say that even this is a great advance in the direction of intellectual culture and refinement. This constitutes a good beginning, an excellent foundation. Create a demand for intellectual culture and the means will soon be supplied. While it is true that the society appears not to have found favor in the eyes of a certain critic, it should be understood that the large majority of its members claim to be pupils not teachers like himself, and few, perhaps, possess the micrographic dictionary, which deficiencies should procure for them a lenient judgment.

Knowing well the majority of the gentlemen having the direction of the organization, we congratulate the society upon the wisdom of its choice, and feel sure that if the *Lens* should not achieve so great a success as we hope for it, the failure will be due neither to lack of talent, culture nor energy on the part of its managers. W. H.

*Plastics and Orthopedics: A Report Republished from the Transactions of the Illinois State Medical Society for* 1871. By DAVID PRINCE, M.D. With numerous wood-cut illustrations.

*The Rejected Address. Man's True Relation to Nature: His Origin, Character and Destiny.* By T. P. WILSON, M.D., Cleveland, Ohio, Editor Ohio Med. and Surg. Reporter. Delivered in Philadelphia, June 6, 1871.

*Artificial Induction of Labor in Uræmia.* By SAMUEL C. BUSEY, M.D., Physician to the Louise Home, etc., etc., Washington, D. C.

